# The IMPACT Survey: the humanistic impact of osteogenesis imperfecta in adults

**DOI:** 10.1186/s12889-024-20555-0

**Published:** 2024-11-28

**Authors:** Taco van Welzenis, Ingunn Westerheim, Tracy Hart, Lena Lande Wekre, Oliver Semler, Frank Rauch, Laetitia Dewavrin, Ruby Dadzie, Samantha Prince, Cathleen Raggio

**Affiliations:** 1 Osteogenesis Imperfecta Federation Europe, Heffen, Belgium; 2 Osteogenesis Imperfecta Foundation, Gaithersburg, MD USA; 3 TRS National Resource Center for Rare Disorders, Sunnaas Rehabilitation Hospital, Nesodden, Norway; 4 Faculty of Medicine and University Hospital Cologne, Department of Pediatrics, University of Cologne, Cologne, Germany; 5 McGill University, Montreal, Canada; 6 Wickenstones Ltd, Oxford, Oxfordshire UK; 7 Hospital for Special Surgery, New York, USA

**Keywords:** Osteogenesis imperfecta, Patient-reported outcomes, Survey, Burden of disease, Fractures, Humanistic burden, Quality of life, Health worries, Pain, Fatigue, Self-reported disease severity

## Abstract

**Background:**

The IMPACT Survey explored the humanistic, clinical, and economic burden of osteogenesis imperfecta (OI) on individuals with OI, their families, caregivers, and wider society. Two previous publications report research methodology, initial insights of the survey, and cost of illness of OI. Here, we present data on the impact of OI on the quality of life (QoL) of adults with OI and explore potential drivers of this impact.

**Methods:**

The IMPACT Survey was an international mixed methods online survey in eight languages (fielded July–September 2021), aimed at adults (aged ≥ 18 years) or adolescents (aged 12–17 years) with OI, caregivers (with or without OI) of individuals with OI, and other close relatives. Survey domains included demographics, socioeconomic factors, clinical characteristics, treatment patterns, QoL, and health economics. We conducted a descriptive analysis of the QoL data, as well as exploratory regression analyses to identify drivers of impact of OI on QoL (independent associations between patient characteristics and the impact on QoL).

**Results:**

1,440 adults with OI participated in the survey. The proportion who reported an impact of OI on their QoL across individual areas in the physical, socioeconomic, and mental well-being domains ranged between 49 and 84%. For instance, 84% of adults reported an impact of OI on the types of leisure activities they could do and 74% on the type of job they could do. More severe self-reported OI and higher fracture frequency were consistently identified as drivers of OI’s impact on QoL. The proportion of adults who reported worrying about different aspects of their lives due to their OI, such as mobility loss, future fractures, and ageing, ranged between 31 and 97%.

**Conclusion:**

IMPACT provides insights into the humanistic burden of OI on adults, revealing that OI has a substantial impact on the QoL of adults. OI severity and fracture frequency were consistently identified as drivers of impact on QoL across all domains. Understanding these drivers may aid in identifying areas for targeted interventions, such as fracture prevention.

**Supplementary Information:**

The online version contains supplementary material available at 10.1186/s12889-024-20555-0.

## Introduction

Osteogenesis imperfecta (OI) is a rare, heritable condition affecting bone and connective tissue structure and function. Its reported incidence is 1/15,000–20,000, although the actual number may be higher [1–4]. While OI is primarily caused by defects in the Type I collagen genes (*COL1A1* and *COL1A2*), it can also be caused by underlying mutations in other genes linked to collagen synthesis, bone mineralisation, or osteoblast differentiation in rare forms of OI [2]. Symptoms of OI include bone fragility, pain, hypermobility, growth defects, dental abnormalities, cardiovascular and pulmonary issues, and hearing loss [2].

Currently, there are no curative treatments for OI. Care approaches aim to improve mobility and independence by alleviating symptoms and helping individuals to manage them in an optimal way. Due to the varying and potentially complex symptoms [5–10] and the ongoing, multidisciplinary management required, living with OI can affect many aspects of a person’s life.

To date, reports on the humanistic impact of OI have been limited, predominantly focusing on children and specific geographic regions [11]. A substantial proportion of the existing literature on the quality of life (QoL) of adults with OI describes physical functioning, with results suggesting that individuals with OI report impaired physical function and independence compared with the overall population [12–32]. Few publications shed light on mental well-being and social functioning; among those that do, certain knowledge gaps persist. Furthermore, research on the effects of OI on mental health reaches conflicting conclusions, with some authors concluding that OI is correlated with mental health problems and others not [13, 16, 17, 19, 20, 23–25, 33, 34]. Few studies investigate social functioning in adults [19, 24, 33–36], hence the need for further research in this area.

The IMPACT Survey was conducted to better understand the humanistic, economic, and clinical impact of OI on individuals and wider society [37, 38]. Westerheim et al. present the overall findings from the survey, notably that irrespective of age, those with OI experienced a multitude of signs, symptoms, and events that impacted their lives. Here, we present the humanistic burden of OI in adults across physical, socioeconomic, and mental well-being QoL domains, and we identify drivers of impact on QoL.

## Methods

### Development

The IMPACT Survey was developed by a steering committee consisting of academic researchers, representatives of the Osteogenesis Imperfecta Foundation (OIF, USA), the umbrella association Osteogenesis Imperfecta Federation Europe (OIFE), and representatives of Mereo BioPharma. The survey prioritised topics identified as evidence gaps in a scoping review [11] that were most relevant to individuals with OI and the research community. For more information on the development, design, and fielding of IMPACT, please refer to Westerheim et al. 2024 [37].

### Survey domains

The survey included questions on demographics, socioeconomic factors, clinical characteristics, treatment patterns, humanistic impact, and economic outcomes [37]. A list of all survey questions and answer options can be found in Supplementary File 1. To investigate humanistic impact, adults with OI were asked questions about the impact of OI on their lives in the past 12 months (response options included a five-level Likert scale [39]: “not impacted”, “very mildly impacted”, “mildly impacted”, “moderately impacted”, and “severely impacted”) and their worries and concerns about the future (response options included a three-level scale: “do not worry”, “worry a little”, and “worry a lot”). These questions covered many aspects of living, such as careers and finances, social life and relationships, physical well-being and functioning, mental well-being, and healthcare (Table 1).

**Table 1:** Domains investigated through questions on quality of life and worries and concerns about the future

Domain	Impact of OI on quality of life ^a^	Worries and concerns ^b^
Physical well-being and functioning	Ability to self-care	Getting older
	Ability to live independently	Ability to care for oneself
	Sexual health	Living independently
		Losing independence
		Losing mobility
		Ability to have a family
		OI complications
		Future fractures
		Pregnancy
		Menopause
Careers and finances	Work hours	Losing a job
	Job type	Future financial situation
	Career choices	Financial means to pay for treatment and care
Social life and relationships	Type of leisure activities	Relationship with family and friends
	Social life	Romantic relationship
	Relationships with family and friends	Future of care recipients with OI
	Romantic relationships	
Mental well-being	Mental health	
	Happiness	
Healthcare		Access to doctors
		Access to medicines
		Access to care
		Side effects of treatment

Abbreviations: OI, osteogenesis imperfecta

^a^Questions 102 & 106 “In the past 12 months, how would you describe the impact that OI has had on your life?”, where respondents received the following instructions: “This question is about understanding the ‘negative’ impacts or challenges you have faced”; ^b^Questions 103, 104, 105, 107, 108, & 109 “Do you feel worried or concerned about any of the following things?”

### Data processing

As previously reported [37], survey data were translated into English and compiled into a master database using the pandas Python software package. Microsoft Excel was used to clean, code and validate data, as well as to generate descriptive statistics. Data were cleaned to exclude any outliers and nonsensical responses. Free text responses were translated into English, and responses were aligned with previous answer options if applicable or categorised into several recurring themes.

### Descriptive analysis

Categorical measures are presented as frequencies (number of respondents, n) and percentages (%) of total survey respondents.

### Regression analysis

Multinomial logistic regression analyses were conducted to identify independent associations, henceforth called drivers, of the impact of OI on QoL (“very mildly”, “mildly”, “moderately”, or “severely” impacted) and worries and concerns (“worry a little” or “worry a lot”). Investigated drivers were self-reported OI severity, sex, age, mobility status, employment status, household living arrangement, fracture frequency in the past 12 months, and clinical signs, symptoms, and events experienced in the past 12 months. Due to a broad alignment found between clinical OI type and self-reported OI severity [37], we did not investigate OI type as a driver. A summary of included and excluded variables can be found in Supplementary Material 2. Results of regression analyses are presented as relative risk ratios (RRR), with p-values ≤ 0.05 considered statistically significant. Regression analyses were performed using R version 4.4.0.

## Results

### Demographics and clinical characteristics

Demographics and clinical characteristics of the adults with OI population (*n* = 1,440) have been previously reported. Briefly, 70% were female, the median age was 43 years, most adults (66%) reported walking unaided, either indoors or outside, and were in paid employment (58%) [37, 38]. The most common household living situation for adults with OI was living with their partner only (29%). We observed variation in reported living and mobility status between those with differing self-reported OI severities; many adults with severe OI reported living alone (32%) or with their parents (32%), whereas two-thirds of adults with mild OI lived with their partner and children if applicable (67%). Most adults with severe OI used wheelchairs indoors or outdoors (83%), and one-fifth (20%) reported walking unaided, whereas only 6% of those with mild OI used wheelchairs, and 95% reported walking unaided (Please see Supplementary Material 3 for a table of demographics of adults with OI).

### Impact of OI on QoL

**Fig. 1:**
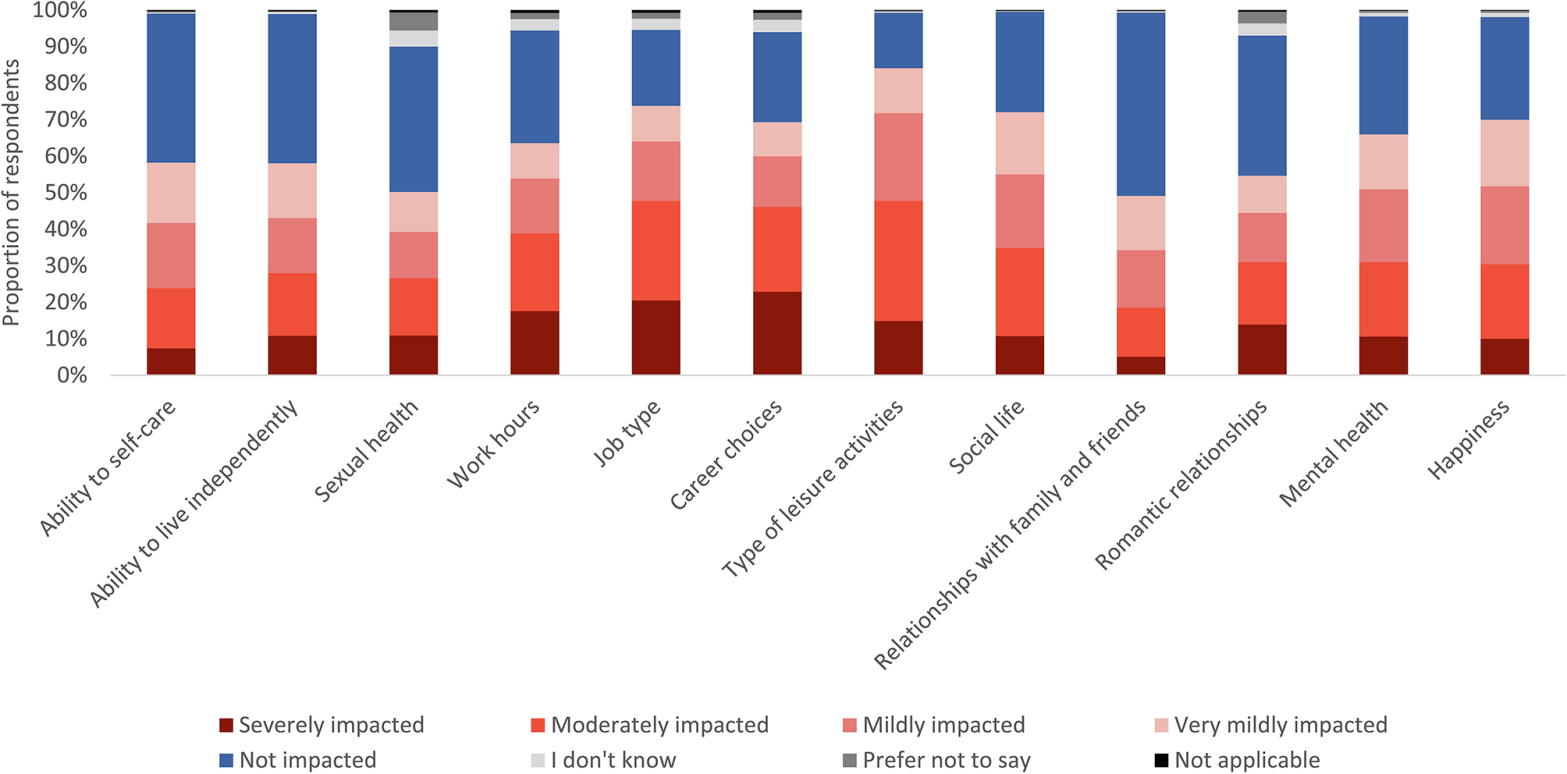
The reported degree of impact of OI on the quality of life of adults with OI, displayed as a stacked bar graph of percentages (n = 1,440). Abbreviations: OI, osteogenesis imperfecta. This graph is based on the responses to questions 102 & 106 “In the past 12 months, how would you describe the impact that OI has had on your life?”, where respondents received the following instructions: “This question is about understanding the ‘negative’ impacts or challenges you have faced”

The proportion of adults whose QoL was impacted is reported as a sum of those impacted very mildly, mildly, moderately, and severely. This grouping encompasses all levels of impact. Across all QoL domains, the proportion of adults who were very mildly impacted represents a similar or smaller proportion of those impacted than those mildly or moderately impacted. These proportions are depicted in Fig. 1. A breakdown of each degree of impact, by different patient characteristics, can be found in Supplementary Material 4.

**Fig. 2:**
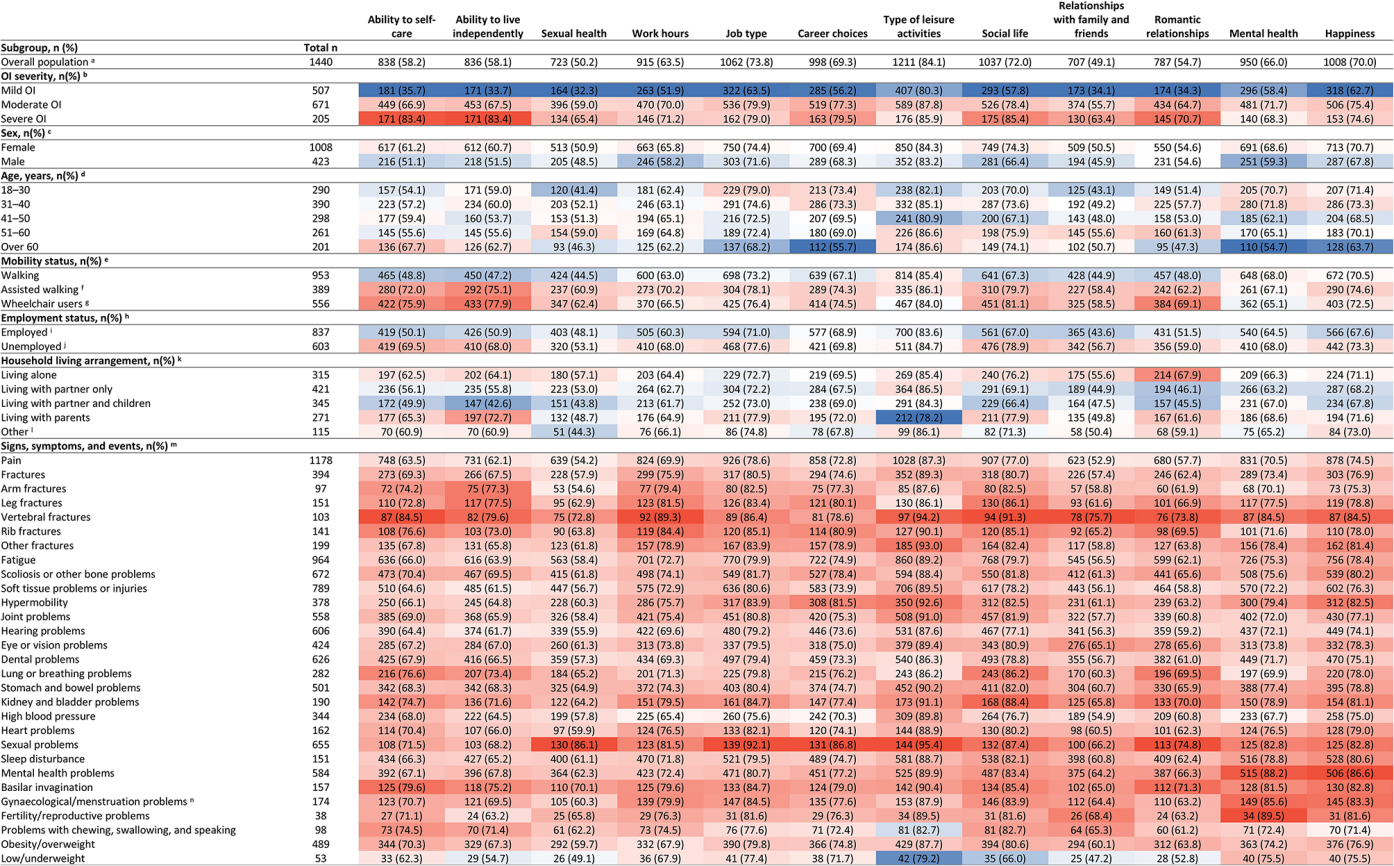
Heatmap of impact of OI (ranging from “very mildly” to “severely” impacted) on quality of life of adults with OI (n = 1,440). Proportions in parentheses are based on category totals. Colours indicate differences to the overall population. Subgroups with similar values to the overall population are shown in white, with greater proportions in red and smaller proportions in blue. Abbreviations: OI, osteogenesis imperfecta. ^a^Questions 102 & 106 “In the past 12 months, how would you describe the impact that OI has had on your life?”, where respondents received the following instructions: “This question is about understanding the ‘negative’ impacts or challenges you have faced”; ^b^Question 18 “How would you describe the severity of your OI?”; ^c^Question 8 “What is your sex?”; ^d^Question 1 “What is your age?”; ^e^Question 16 “How do you get around”; ^f^“Assisted walking” includes the use of walking sticks/canes, walking frames, rollators or crutches; ^g^“Wheelchair users” includes the use of manual wheelchairs, powered wheelchairs or mobility scooters; ^h^Question 9 “Please indicate which of the following best describe you”; ^i^“Employed” includes respondents who answered “I am in paid employment/self-employed”; ^j^“Unemployed” includes retirees, students, homemakers and volunteers; ^k^Questions 11 & 12 “Who do you live with?“, multiple answer options possible; ^l^“Other” includes single parents, living with friends or house share, living with caregiver or assistant, and living in supported living accommodation or a care home; ^m^Question 113 “Over the past 12 months, have you experienced any of the following signs, symptoms, or events?“; ^n^Population potentially affected by gynaecological/menstruation problems include female respondents only

Across all 12 QoL areas queried (Table 1), OI had an impact (ranging from very mild to severe) on the lives of adults. At least 49% of adults with OI were affected in each area. The areas most commonly impacted by OI (reported by over 70% of participants) were job types (74%), social life (72%), and types of leisure activities respondents could do (84%). Areas less commonly impacted were sexual health and relationships with family and friends, yet a substantial proportion of adults were impacted in these areas (50% and 49%, respectively; Table 2; Figs. 1 and 2, Supplementary Material 4).

**Table 2:** Impact of OI on quality of life of adults with OI (*n* = 1,440)

Impact on quality of life, *n* (%) ^a^	Severely impacted	Moderately impacted	Mildly impacted	Very mildly impacted	Not impacted	I don’t know	Prefer not to say	Not applicable
Ability to self-care	106 (7.4)	237 (16.5)	258 (17.9)	237 (16.5)	588 (40.8)	4 (0.3)	5 (0.3)	5 (0.3)
Ability to live independently	156 (10.8)	246 (17.1)	218 (15.1)	216 (15.0)	588 (40.8)	8 (0.6)	3 (0.2)	5 (0.3)
Sexual health	157 (10.9)	225 (15.6)	183 (12.7)	158 (11.0)	573 (39.8)	63 (4.4)	72 (5.0)	9 (0.6)
Work hours	253 (17.6)	307 (21.3)	216 (15.0)	139 (9.7)	444 (30.8)	45 (3.1)	24 (1.7)	12 (0.8)
Job type	295 (20.5)	392 (27.2)	235 (16.3)	140 (9.7)	300 (20.8)	44 (3.1)	23 (1.6)	11 (0.8)
Career choices	329 (22.8)	335 (23.3)	199 (13.8)	135 (9.4)	355 (24.7)	49 (3.4)	27 (1.9)	11 (0.8)
Type of leisure activities	214 (14.9)	473 (32.8)	346 (24.0)	178 (12.4)	218 (15.1)	4 (0.3)	4 (0.3)	3 (0.2)
Social life	154 (10.7)	348 (24.2)	290 (20.1)	245 (17.0)	396 (27.5)	2 (0.1)	2 (0.1)	3 (0.2)
Relationships with family and friends	73 (5.1)	194 (13.5)	226 (15.7)	214 (14.9)	723 (50.2)	4 (0.3)	3 (0.2)	3 (0.2)
Romantic relationships	200 (13.9)	246 (17.1)	194 (13.5)	147 (10.2)	552 (38.3)	48 (3.3)	46 (3.2)	7 (0.5)
Mental health	153 (10.6)	293 (20.3)	287 (19.9)	217 (15.1)	465 (32.3)	14 (1.0)	8 (0.6)	3 (0.2)
Happiness	144 (10.0)	294 (20.4)	307 (21.3)	263 (18.3)	404 (28.1)	17 (1.2)	8 (0.6)	3 (0.2)

Abbreviations: OI, osteogenesis imperfecta

^a^Questions 102 & 106 “In the past 12 months, how would you describe the impact that OI has had on your life?”, where respondents received the following instructions: “This question is about understanding the ‘negative’ impacts or challenges you have faced”

Among those with severe OI, at least 65% of adults were affected in each QoL area investigated. Compared with the overall cohort of adults with OI, a greater proportion of those with severe OI were severely impacted by their OI across QoL areas. OI impacted the romantic relationships of 37% of adults with severe OI, compared to 5% of the overall cohort of adults with OI (Fig. 2, Supplementary Material 5).

Some adults provided free-text responses on the positive impacts of OI on their lives, including developing positive personality traits, such as being more empathetic or resilient and feeling a sense of community.

### Drivers of impact of OI on QoL

In each QoL domain (Table 1), several demographics and clinical characteristics were identified as drivers, though not all were common across all domains or levels of impact (graphs showing the results from the regression analysis can be found in Supplementary Material 6, 7, 8 and 9, and relative risk ratios in Supplementary Material 10). However, we observed trends across QoL domains, where certain demographics or clinical characteristics were consistently identified as drivers of impact on QoL.

### Drivers of impact on physical well-being and functioning

Various drivers were identified for impact of OI on physical QoL (Supplementary Material 6A–H). Self-reported OI severity, mobility status, and fracture frequency were consistently identified as drivers of OI impact on physical QoL across varying degrees of impact. For example, adults with self-reported moderate or severe OI were more likely to experience a mild to severe impact on their physical health compared with adults with mild OI; those with severe OI were 12.6 times (RRR, *P* < 0.001) more likely to experience a severe impact on their ability to self-care than those with mild OI (Supplementary Material 6A). Mobility status was also identified as a driver of ability to self-care. Individuals who used mobility aids were 1.5 times (RRR, *P* < 0.05) more likely to experience a moderate impact on their ability to self-care than those who did not, and those who used wheelchairs were 2.6 times (RRR, *P* < 0.05) more likely to experience a severe impact on their ability to self-care than those who did not (Supplementary Material 6F). Additionally, those who experienced multiple fractures were more likely to experience a severe impact on their physical health than those who did not fracture. For instance, adults who fractured twice (RRR 3.6, *P* < 0.05) or more often (RRR 3.9, *P* < 0.001) in the past 12 months were more likely to experience a severe impact on their sexual health than those who did not fracture (Supplementary Material 6D).

### Drivers of impact on social life and relationships

Self-reported OI severity was consistently identified as a driver of impact on social QoL (Supplementary Material 7A). For instance, individuals with severe OI were 16.5 times (RRR, *P* < 0.001) more likely to experience a severe impact on social life than those with mild OI. Adults over the age of 30 were more likely to report a moderate impact on relationships with family and friends compared with 18- to 30-year-olds, and female respondents were less likely (RRR 0.6, *P* < 0.05) to experience a severe impact on their romantic relationships than male respondents (Supplementary Material 7B–C). Nonetheless, the impact of age and sex were inconsistent across social QoL areas.

### Drivers of impact on careers and finances

Self-reported OI severity and higher fracture frequency were consistently identified as drivers of impact on careers (Supplementary Material 8A and D). For instance, those who experienced three or more fractures were more likely to report any degree of impact on the number of hours they could work than those who did not fracture (Supplementary Material 8D). Furthermore, individuals who experienced pain were 4.1 (RRR, *P* < 0.001) times more likely to report a severe impact on their work hours than those who did not (Supplementary Material 8E).

### Drivers of impact on mental health and well-being

In line with findings across other QoL domains, self-reported OI severity and fracture frequency were consistently identified as drivers of OI impact on mental health and happiness (Supplementary Material 9A and D). For instance, those who experienced three or more fractures were 2.6 times (RRR, *P* < 0.05) more likely to experience a severe impact on their mental health than those who did not fracture (Supplementary Material 9D). Female respondents were 1.6 times (RRR, *P* < 0.05) more likely to experience a moderate impact on mental health than male respondents. Other levels of impact (“severely”, “mildly”, and “very mildly” impacted) were comparable between male and female respondents (Supplementary Material 9C).

### OI-linked worries and concerns

**Fig. 3:**
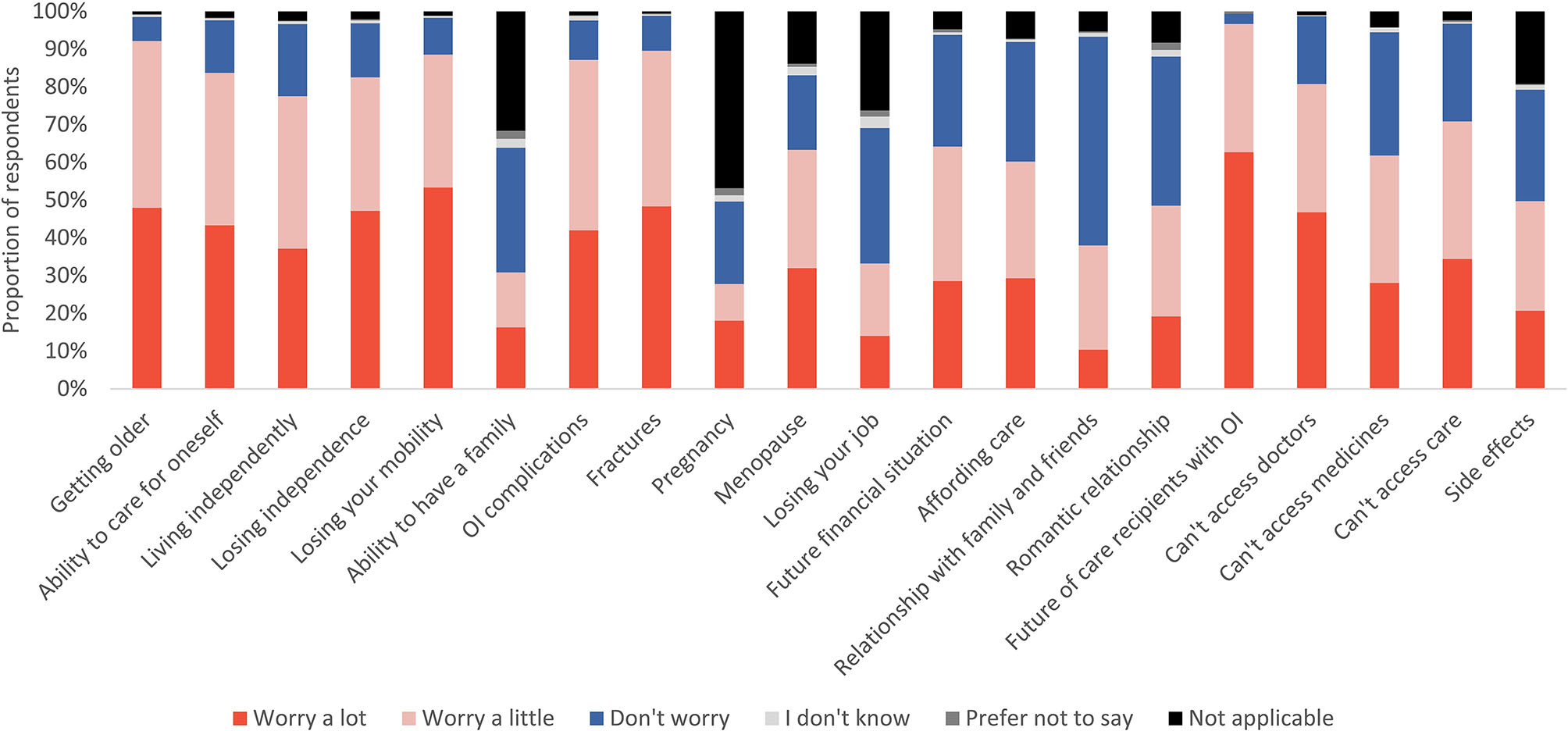
The reported degree of worry about the listed circumstances of adults with OI, displayed as a stacked bar graph of percentages (*n* = 1,440). Abbreviations: OI, osteogenesis imperfecta. This graph is based on the responses to questions 103, 104, 105, 107, 108, & 109 “Do you feel worried or concerned about any of the following things?”. The answer options “Living independently” and “Ability to have a family” were only given to adults with OI who did not have any care recipients with OI (*n* = 1,290). Population potentially affected by pregnancy and menopause include female respondents only (*n* = 1,008). The answer option “Worrying about future of care recipients with OI” was only given to adults with OI who are caregivers for individuals with OI (*n* = 150)

**Fig. 4:**
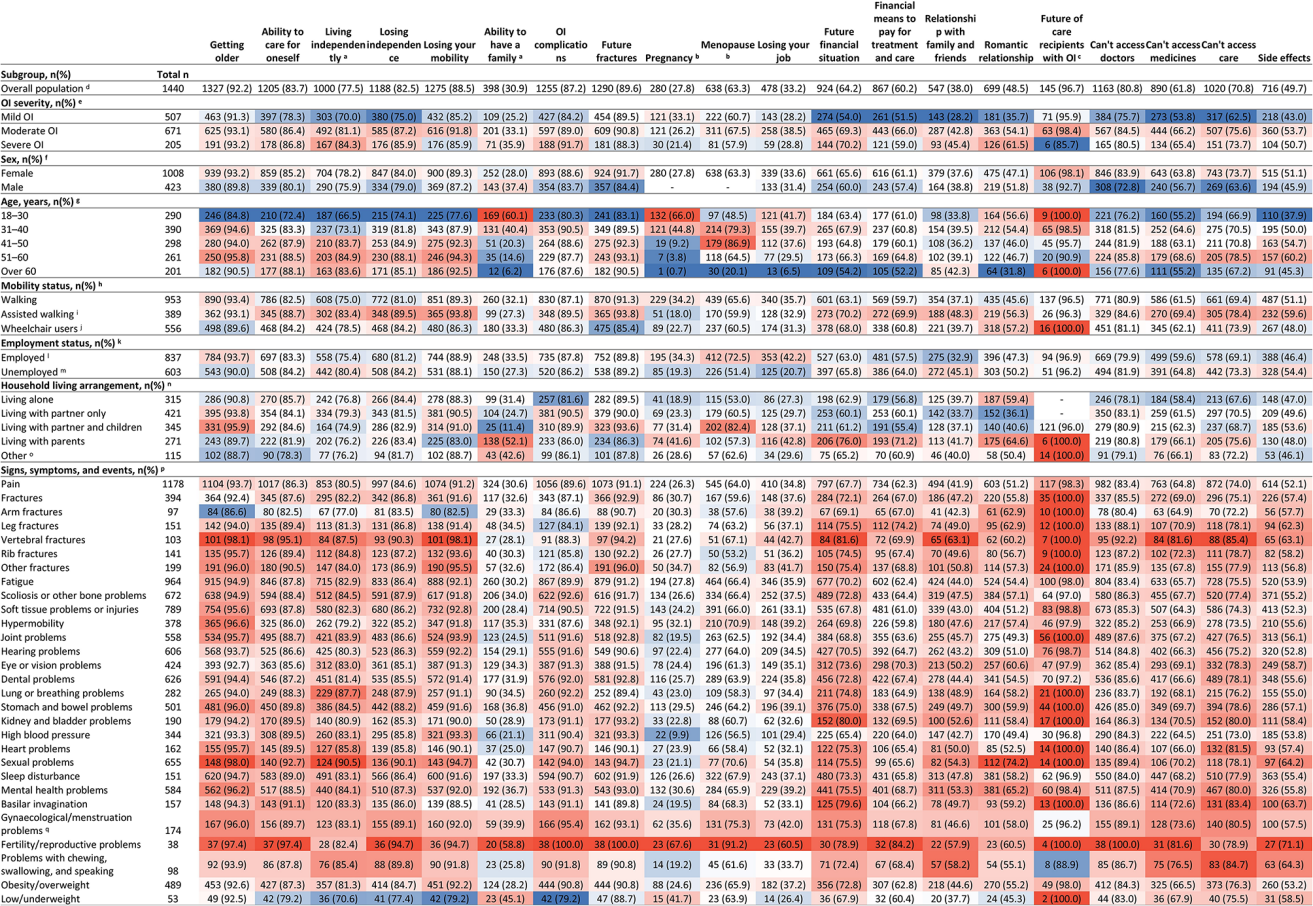
Heatmap showing proportion of adults who worry a lot or a little about the listed circumstances (*n* = 1,440). Proportions in parentheses are based on category totals. Colours indicate differences to the overall population. Subgroups with similar values to the overall population are shown in white, with greater proportions in red and smaller proportions in blue. Abbreviations: OI, osteogenesis imperfecta. ^a^Only adults without care recipients with OI (*n* = 1,290) were provided with the answer options “Living independently” and “Ability to have a family”; ^b^Population potentially affected by pregnancy and menopause include female respondents only (*n* = 1,008); ^c^Only caregivers with OI (*n* = 150) were provided with the answer option “Worrying about future of care recipients with OI”; ^d^Questions 103, 104, 105, 107, 108 & 109 “Do you feel worried or concerned about any of the following things?”; ^e^Question 18 “How would you describe the severity of your OI?”; ^f^Question 8 “What is your sex?”; ^g^Question 1 “What is your age?”; ^h^Question 16 “How do you get around”; ^i^“Assisted walking” includes the use of walking sticks/canes, walking frames, rollators or crutches; ^j^“Wheelchair users” includes those who use manual wheelchairs, powered wheelchairs or mobility scooters; ^k^Question 9 “Please indicate which of the following best describe you”; ^l^“Employed” includes respondents who answered “I am in paid employment/self-employed”; ^m^“Unemployed” includes retirees, students, homemakers and volunteers; ^n^Questions 11 & 12 “Who do you live with?”, multiple answer options possible; ^o^“Other” includes single parents, living with friends or house share, living with caregiver or assistant, and living in supported living accommodation or a care home; ^p^Question 113 “Over the past 12 months, have you experienced any of the following signs, symptoms, or events?”; ^q^Population potentially affected by gynaecological/menstruation problems include female respondents only

The degree to which adults with OI worried varied depending on the circumstance queried (Table 3). Over half of adults worried a lot about mobility loss (53%), future fractures (48%), and ageing (48%). Areas of lower concern included relationships with family and friends (10%) and job security (14%; Table 3; Figs. 3 and 4, Supplementary Material 11).

**Table 3:** Degree of worry about the listed circumstances of adults with OI (*n* = 1,440)

Degree of worry, *n* (%) ^a^	Worry a lot	Worry a little	Don’t worry	Not applicable	I don’t know	Prefer not to say
Getting older	690 (47.9)	637 (44.2)	91 (6.3)	11 (0.8)	9 (0.6)	2 (0.1)
Ability to care for oneself	624 (43.3)	581 (40.3)	201 (14.0)	24 (1.7)	7 (0.5)	3 (0.2)
Living independently ^b^	479 (37.1)	521 (40.4)	246 (19.1)	32 (2.5)	9 (0.7)	3 (0.2)
Losing independence	679 (47.2)	509 (35.3)	206 (14.3)	30 (2.1)	11 (0.8)	5 (0.3)
Losing mobility	768 (53.3)	507 (35.2)	140 (9.7)	16 (1.1)	7 (0.5)	2 (0.1)
Ability to have a family ^b^	210 (16.3)	188 (14.6)	426 (33.0)	408 (31.6)	30 (2.3)	28 (2.2)
OI complications	604 (41.9)	651 (45.2)	150 (10.4)	15 (1.0)	17 (1.2)	3 (0.2)
Future fractures	696 (48.3)	594 (41.3)	133 (9.2)	8 (0.6)	5 (0.3)	4 (0.3)
Pregnancy ^c^	182 (18.1)	98 (9.7)	220 (21.8)	472 (46.8)	17 (1.7)	19 (1.9)
Menopause ^c^	322 (31.9)	316 (31.3)	199 (19.7)	140 (13.9)	23 (2.3)	8 (0.8)
Losing a job	202 (14.0)	276 (19.2)	517 (35.9)	378 (26.3)	44 (3.1)	23 (1.6)
Future financial situation	411 (28.5)	513 (35.6)	426 (29.6)	68 (4.7)	10 (0.7)	12 (0.8)
Financial means to pay for treatment and care	422 (29.3)	445 (30.9)	456 (31.7)	104 (7.2)	9 (0.6)	4 (0.3)
Impact on relationships with family and friends	149 (10.3)	398 (27.6)	796 (55.3)	77 (5.3)	15 (1.0)	5 (0.3)
Impact on romantic relationships	276 (19.2)	423 (29.4)	568 (39.4)	119 (8.3)	26 (1.8)	28 (1.9)
Future of care recipients with OI ^d^	94 (62.7)	51 (34.0)	4 (2.7)	0 (0.0)	0 (0.0)	1 (0.7)
Access to doctors	673 (46.7)	490 (34.0)	258 (17.9)	15 (1.0)	4 (0.3)	0 (0.0)
Access to medicines	404 (28.1)	486 (33.8)	470 (32.6)	61 (4.2)	18 (1.3)	1 (0.1)
Access to care	495 (34.4)	525 (36.5)	373 (25.9)	34 (2.4)	7 (0.5)	6 (0.4)
Side effects of treatment	298 (20.7)	418 (29.0)	425 (29.5)	277 (19.2)	18 (1.3)	4 (0.3)

Abbreviations: OI, osteogenesis imperfecta

^a^Questions 103, 104, 105, 107, 108, & 109 “Do you feel worried or concerned about any of the following things?“; ^b^These answer options were only given to adults with OI who did not have any care recipients with OI (*n* = 1,290); ^c^Population potentially affected by pregnancy and menopause include female respondents only (*n* = 1,008); ^d^This answer option was only given to adults with OI who are caregivers for individuals with OI (*n* = 150)

Similar proportions of those with severe OI worried about various aspects of living compared with the overall cohort of adults with OI. However, smaller proportions of adults with severe OI worried about pregnancy (21% vs. 28% of the overall cohort) or the future of their care recipients with OI (86% of caregivers with severe OI vs. 97% of the overall cohort of caregivers). Greater proportions of adults with severe OI worried about their romantic relationships (61%) compared with the overall cohort of adults with OI (49%; Fig. 4, Supplementary Material 12).

### Drivers of worries and concerns

Various drivers were identified for worrying about different areas of respondents’ lives (graphs showing the results of the regression analysis can be found in Supplementary Material 13, 14, 15, and 16 and relative risk ratios in Supplementary Material 17). As was the case for QoL, self-reported OI severity was also identified as a driver of OI-linked worries (“worry a little” or “worry a lot”). Across various areas of their lives, adults with self-reported moderate or severe OI were more likely to report worrying than those with mild OI. For example, those with severe OI were 5.8 (RRR, *P* < 0.001) times more likely to worry a lot about getting older and 8.6 (RRR, *P* < 0.001) times more likely to worry a lot about their romantic relationships than those with mild OI (Supplementary Material 13A and 14A). Compared to 18- to 30-year-olds, older respondents were more likely to worry (“a lot” and “a little”) about physical well-being and functioning (Supplementary Material 13B).

## Discussion

With data compiled from 1,440 adults, the IMPACT Survey is the most extensive patient-reported dataset on the experience of individuals with OI to date. This report provides insights into the humanistic impact of OI on adults across physical, socioeconomic, and mental well-being QoL domains. Irrespective of demographic and clinical characteristics, most adults experienced an impact on their lives and were worried or concerned about their future.

### Impact of OI on QoL across all areas

Sex, mobility status, household living arrangement, employment status, and clinical signs, symptoms, and events were significantly associated with impact on various areas of QoL. However, there was no clear trend: these drivers were not consistently significantly associated across multiple degrees of impact or QoL domains. On the other hand, we found that self-reported OI severity and fracture frequency were consistent drivers of impact on QoL across multiple degrees of impact and QoL domains.

Previous studies identified a correlation between OI severity and QoL impairment, where increased OI severity was correlated with lower QoL [13, 40]. These findings align with the findings of the present study. Additionally, previous studies report that fracture rates are higher in children than in adults with OI [41] but that fracture incidence rates in adults with OI [42] remain higher than in the general population [41, 43]. In adults with OI, fractures impact daily life and emotional well-being [44], with widespread and long-lasting effects on QoL [27, 45]. Fractures can also impair physical function [27], especially in individuals who use mobility aids or those with more severe OI [46]. Our findings suggest that the use of mobility aids and wheelchairs is a driver of impact on ability to self-care. They also suggest that OI severity is a driver of impact on QoL across all areas. Notably, the impact of fractures can extend past physical effects. In previous research, children and adults with OI have reported anxiety about future fractures [47]. This aligns with the results from our study, which found that 90% of adults worried about fractures “a lot” or “a little”.

### Impact of OI on the ability to self-care

Over half of the respondents (58%) reported that OI impacted their ability to self-care (ranging from “very mild” to “severe” impact, with 24% reporting a moderate to severe impact). Older individuals in the general population often report a diminished ability to self-care [48, 49]; studies link the ability to self-care to life satisfaction or happiness [50, 51]. In our study, both younger and older adults reported that their ability to self-care was impacted. In addition, similar proportions of younger and older adults worried (either “a lot” or “a little”) about their ability to self-care, highlighting the challenges faced by adults with OI over their lifetimes. Deterioration of an individual’s ability to self-care may happen earlier in life for individuals with OI compared with the general population. These effects may be exacerbated in individuals with OI who use mobility aids, as supported by our findings, which suggest that the use of mobility aids and wheelchairs are drivers of impact on ability to self-care.

### Impact of OI on social life and relationships

Around half of adults reported an impact (ranging from “very mild” to “severe” impact) on their relationships with family and friends (49%) and romantic relationships (55%). Furthermore, many adults worried (either “a lot” or “a little”) about their relationships with family and friends (38%) and romantic relationships (49%). Strong interpersonal relationships have been shown to play a crucial role in the overall well-being of the general population [52]. Additionally, social support has been linked to well-being in individuals with chronic illnesses [53]. Chronic illness can strain relationships for various reasons [54] and may affect family dynamics [55]. Individuals with OI may face physical limitations hindering social activities due to health issues linked to OI, such as fractures and hearing loss. Studies have also identified a relationship between disabling barriers and loneliness or social isolation among people with disabilities [56–59]. In addition to the effects of disability, some groups, such as men and older adults, are more likely to experience loneliness [60] and less likely to form intimate friendships [61]. Similarly, our study found that adults with more severe OI, males and older adults (over 60 years) were more likely to experience a severe impact on their relationships. Past research has found worse QoL in older adults with OI [12, 17, 62].

### Impact of OI on careers and finances

Over 60% of the adults in our survey reported an impact on their careers and finances. We previously reported that adults with OI missed 1.7 workdays (average) and spend €191 out-of-pocket (average over categories including medicine, physiotherapy, and personal care) over 4 weeks due to their OI [38]. In this report, we identified fractures and pain as drivers of impact on careers. Previous studies have found that chronic pain can negatively impact careers. For example, chronic pain was found to be associated with reduced work performance and negatively correlated with employment retention [63, 64]. Pain is commonly experienced by individuals with OI and can affect QoL [17, 33, 34, 62, 65]. Experiencing fractures can also affect careers among the general population by physically hindering individuals’ ability to work, resulting in lost work hours and, in some cases, preventing a return to work after fracturing [66, 67].

### Impact of OI on mental health

In our survey, 66% of adults reported that OI impacts their mental health (ranging from “very mild” to “severe” impact, with 30% reporting a moderate to severe impact). In the IMPACT Survey sample, 40% of participants reported experiencing mental health problems in the past 12 months. Since our survey was fielded in 2021, the COVID-19 pandemic may have resulted in respondents reporting a greater prevalence of mental health problems compared with other years, as was observed in the general population [68].

Despite the impact of the pandemic on mental health, a prevalence of around 40% is in line with the literature on mental health in chronic illness. One review of 29 studies representing 16,000 people with diabetes, obesity, cancer, COPD, or heart disease estimated that the prevalence of anxiety or depression among individuals with chronic illnesses was 36.6% [69]. Furthermore, previous studies have found associations between mental health problems and chronic illness [70]. While OI impacted the mental health of most adults in our survey, some individuals were more impacted than others. Mental health problems were commonly reported by those who experienced any fractures (73%) or vertebral fractures (84%) in the past 12 months.

### Impact of OI on attitudes and outlook on life

Our study, like others on chronic illnesses, may not fully reflect the true impact of OI on QoL. Previous reports indicate that symptom underreporting, which may be influenced by factors such as stigma and denial [71, 72], could affect the reported impact of chronic illnesses on QoL. Additionally, studies have found that living with a chronic illness may build resilience and impart a tendency towards stoicism [73]; both are positively associated with better QoL [74, 75]. Resilience was reported in free text responses in our study, demonstrating the adversity that individuals with OI may have to cope with. Some adults also reported an increased sense of empathy and positive impacts on their social life and careers when they found friends, partners, or career opportunities through OI networks. Participation in patient networks was reported to impact QoL positively, through community events and support groups, which highlights the importance of patient groups.

### Implications and future research directions

Particular attention needs to be paid to the broader implications of fractures on individuals with OI to improve their QoL. By focusing resources on fracture prevention and pain relief, the careers and productivity of adults with OI may be improved. Furthermore, workplace accommodations, such as the option to work remotely, could help to reduce the negative impact of OI on careers. Working remotely has previously been reported to have a positive impact on well-being for people with disabilities [76]. While OI is associated with a significant impact on various aspects of adults’ lives, there remains a need for more nuanced understanding of the factors driving its impact on QoL. Further studies that collect data on a representative sample from lower-income countries will help address the relationship between poverty and the impact of OI on QoL. Exploring the underlying factors influencing independence and the ability to self-care would provide a more comprehensive understanding of how to address the impact of OI on QoL. Investigating additional potential drivers of impact on QoL, such as economic status, may help identify areas for targeted interventions to improve the lives of adults with OI. This will be aided by a better understanding of the complex relationship between OI and QoL.

### Strengths and limitations

Our survey has collated the largest sample of respondents with OI and their caregivers to date [37], and it covers a breadth of topics that have not been previously explored in such detail. To ensure that the survey explored topics of interest to the community, this survey was conducted with input from OI experts and members of the OI community. QoL questions were bespoke to the survey and were reviewed by OI experts and members of the OI community.

The impact of COVID-19 may have led to more negative responses about QoL, worries and concerns. Among the general population, the pandemic and lockdowns affected the social life, leisure activities, careers, and mental health of many individuals [77]. The OI population reported heightened anxiety due to the pandemic [78].

Our survey did not collect data from the general population, which precludes comparison. Direct age-matched controls would improve the reliability of QoL research. Furthermore, our study cohort is not representative of the global OI population. This is due to increased engagement of certain groups, such as females, individuals with more severe OI, and individuals from Europe and the United States. This is typically seen in other surveys. However, despite the rarity of the condition, a large number of respondents engaged with the survey, enabling the collection of a substantial amount of data on underrepresented groups.

This work did not use any validated QoL tools, which may affect the validity of responses. However, since OI is a rare disease, using validated tools may have led to an incomplete understanding of the disease burden by not capturing the unique aspects of the impact of OI on QoL. Recall bias may have also affect our results, as the survey collected self-reported data. However, data on QoL reported by individuals with OI provides unique and specific insights into the experience of those living with OI.

## Conclusion

Data from the IMPACT Survey suggest that OI strongly impacts the lives of adults across all investigated QoL domains (physical, socioeconomic, and mental well-being), regardless of demographics and clinical characteristics. Across several areas, more severe self-reported OI and increased fracture frequency were identified as drivers of impact of OI on QoL and OI-linked worries and concerns. Fractures impact the QoL of adults with OI, which highlights the need for improved prevention efforts in adults.

## Electronic supplementary material

Below is the link to the electronic supplementary material.

Supplementary Material 1

Supplementary Material 2

Supplementary Material 3

Supplementary Material 4

Supplementary Material 5

Supplementary Material 6

Supplementary Material 7

Supplementary Material 8

Supplementary Material 9

Supplementary Material 10

Supplementary Material 11

Supplementary Material 12

Supplementary Material 13

Supplementary Material 14

Supplementary Material 15

Supplementary Material 16

Supplementary Material 17

## Data Availability

Relevant data is provided within the manuscript or supplementary information files. Further data that support the findings of this study are not openly available due to reasons of sensitivity. They are managed by a data management committee and are available upon reasonable request to the authors (impactsurvey@wickenstones.com). Specific data requests can be made on the IMPACT Survey website: https://www.impactsurveyoi.com/.
